# Public awareness and individual responsibility needed for judicious use of antibiotics: a qualitative study of public beliefs and perceptions

**DOI:** 10.1186/s12889-018-6047-8

**Published:** 2018-10-03

**Authors:** Mirko Ancillotti, Stefan Eriksson, Jorien Veldwijk, Jessica Nihlén Fahlquist, Dan I. Andersson, Tove Godskesen

**Affiliations:** 1 Centre for Research Ethics & Bioethics, Department of Public Health and Caring Sciences, Uppsala University, Box 564, 751 22 Uppsala, Sweden; 2 Institute of Health Policy and Management, Erasmus University, Bayle (J) building - Campus Woudestein, Burgemeester Oudlaan 50, 3062 PA Rotterdam, The Netherlands; 3 Department of Medical Biochemistry and Microbiology, Uppsala University, Box 582, 751 23 Uppsala, Sweden; 4 Department of Health Care Sciences, Ersta Sköndal Bräcke University College, Ersta Sköndal Bräcke högskola, Stigbergsgatan 30, 100 61 Stockholm, Sweden

**Keywords:** Antibiotic resistance, Health belief model, Health behavior, Qualitative research

## Abstract

**Background:**

High consumption of antibiotics has been identified as an important driver for the increasing antibiotic resistance, considered to be one of the greatest threats to public health globally. Simply informing the public about this consequence is insufficient to induce behavioral change. This study explored beliefs and perceptions among Swedes, with the aim of identifying factors promoting and hindering a judicious approach to antibiotics use. The study focused primarily on the medical use of antibiotics, also considering other aspects connected with antibiotic resistance, such as travelling and food consumption.

**Methods:**

Data were collected through focus group discussions at the end of 2016. Twenty-three Swedes were recruited using an area-based approach and purposive sampling, aiming for as heterogeneous groups as possible regarding gender (13 women, 10 men), age (range 20–81, mean 38), and education level. Interview transcripts were analyzed using qualitative content analysis. The Health Belief Model was used as a theoretical framework.

**Results:**

Antibiotic resistance was identified by participants as a health threat with the potential for terrible consequences. The severity of the problem was perceived more strongly than the actual likelihood of being affected by it. Metaphors such as climate change were abundantly employed to describe antibiotic resistance as a slowly emerging problem. There was a tension between individual (egoistic) and collective (altruistic) reasons for engaging in judicious behavior. The individual effort needed and antibiotics overprescribing were considered major barriers to such behavior. In their discussions, participants stressed the need for empowerment, achieved through good health communication from authorities and family physicians.

**Conclusions:**

Knowledge about antibiotic consumption and resistance, as well as values such as altruism and trust in the health care system, has significant influence on both perceptions of individual responsibility and on behavior. This suggests that these factors should be emphasized in health education and health promotion. To instead frame antibiotic resistance as a slowly emerging disaster, risks diminish the public perception of being susceptible to it.

**Electronic supplementary material:**

The online version of this article (10.1186/s12889-018-6047-8) contains supplementary material, which is available to authorized users.

## Background

Antibiotics are used to treat many community- and hospital-acquired bacterial infections. They are considered cornerstones of modern medicine in that they are needed to prevent and treat infections associated with, inter alia, cancer and burn treatment, chronic diseases, device surgery, transplantations and neonatal care [1]. Antibiotic resistance (AR) is a form of drug resistance where bacteria can survive exposure to antibiotics. It is an inevitable process, which is speeded up due to human behavior, as the mere usage of antibiotics enriches and selects for resistance in humans, animals, and the environment. The World Health Organization (WHO) regards the rapid development of multidrug resistant (MDR) bacteria as one of the most significant threats to public health globally, as it severely restricts the possibility of treating infectious diseases [2].

To curb AR, a strategic objective is to improve public awareness and understanding, WHO says. Other strategic objectives are to strengthen the knowledge and evidence base, to reduce the incidence of infection, to optimize the use of antimicrobial medicines in human and animal health, and to develop the economic case for sustainable investment [2]. Improving public awareness and understanding depends on effective communication, education and training. Studies suggest that improved understanding of antibiotics may make people feel and act more responsibly [3], but, although being a prerequisite for judicious behavior, information-giving in itself might be insufficient to change behavior [4]. Investigating the perceptions, beliefs and, ultimately, the public health behavior influencing AR is crucial to identify where and how to intervene in health education and health promotion [5].

Containment of AR requires both local and global strategies to improve public awareness and understanding. We work from the premises that local strategies can benefit from the investigation of the beliefs and perceptions that influence AR and that such investigations should be sensitive to context and culture. Possible solutions which may help reducing AR in a country or population may have limited effects in others. Nonetheless, the knowledge generated can of course still be of use in other contexts.

To explore antibiotics-related beliefs and perceptions in a country (Sweden), where the public have some knowledge of AR and comparatively use antibiotics more responsibly, might increase the understanding of factors behind judicious and non-judicious approaches to antibiotics. In Europe, AR varies widely and is generally higher in southern and south-eastern Europe than in the north. In Sweden, consumption of oral antibiotics is lower than in other European states and the population knows comparatively more about AR and use antibiotics more judiciously [6]. Local and national cooperation characterizes Swedish work on containment of AR and since 1989 there are County Medical Officers for communicable disease control. The Swedish Strategic Program against Antibiotic Resistance (Strama), whose overall aim is to preserve antibiotics effectiveness, has worked at regional and national levels since 1994. Starting in 2000 (and extended in 2012), a plan for coordinated work towards the containment of antibiotic resistance and healthcare-associated diseases is jointly run by the National Board of Health and Welfare and the Swedish Board of Agriculture. As a result of such early commitments to curb AR, antibiotic consumption started to decrease already in the 90s [7].

The present study aimed to explore Swedes’ beliefs about and perceptions of antibiotics, in order to find which factors promote or hinder a judicious approach to antibiotics. To our knowledge, while a few studies have quantitatively investigated the Swedish population [8, 9], and Swedish travelers qualitatively [10, 11], no previous qualitative research has been done on public perception and belief affecting antibiotics-related health behavior.

## Methods

### Design

A qualitative and explorative design was used to collect data through focus group discussions (FGDs). FGDs provide insight into behavior by generating a process that helps participants to self-disclose [12, 13].

The Health Belief Model (HBM) was used for developing the interview guide, in the data analysis and the discussion of the results. HBM is a psychological, theoretical model extensively used to explain changes in and maintenance of health-related behavior, not least for infectious disease studies [14, 15]. According to the HBM, personal demographic and psychological characteristics influence how people perceive the seriousness of and susceptibility to a disease, as well as barriers to and benefits of treatment. Each of these tenets can contribute to an explanation of health behavior. By weighing these health beliefs against possible cues for action and the individual’s perceived self-efficacy, an understanding of health behavior can be achieved [16, 17]. Table 1 describes how HBM constructs were applied to this study.

**Table 1:** Application of Health Belief Model Constructs to antibiotic issues

Construct	Application
Perceived susceptibility	The participant’s subjective assessment/perception of the likelihood of being personally affected by antibiotic-resistant bacteria
Perceived seriousness	The participant’s assessment/perception of the severity of the situation regarding antibiotic-resistant bacteria
Perceived benefits	The participant’s assessment/perception of the benefits of engaging in judicious behavior in relation to antibiotics
Perceived barriers	The participant’s assessment/perception of barriers to engaging in judicious behavior in relation to antibiotics
Perceived self-efficacy	The participant’s perception of his/her or others’ competence in engaging in judicious behavior in relation to antibiotics
Cues to action	Trigger mechanisms to prompt engagement in judicious behavior in relation to antibiotic use

The interview guide was structured according to state of the art guidelines for focus groups [12]. The guide was developed by the authors for this study and it was based on a review of the existing literature investigating antimicrobial-related awareness, knowledge, attitudes, beliefs, and behavior.

The structure and themes of the interview were the following: A) Opening question (introducing oneself and reasons for participating); B) Introductory question (spontaneous thoughts about antibiotics); C) Transition question (personal experience of antibiotics); D) Key questions (I - advantages and disadvantages of using antibiotics, II – prescriptions, III - consequences in the present and in the future of misuse of antibiotics at individual and community level, IV - AR, V - individual responsibility, commitment and cues to action); E) Ending question (imagining to advise health authorities) (see Additional file 1).

Follow-up and probing questions were used for clarification and elaboration. The research team thoroughly discussed the interview guide, and after a pilot study, a few questions were eliminated to reduce participants’ fatigue while key questions were arranged in a more consequent order.

### Sampling, recruitment & data collection

Participants were recruited from the general population. Inclusion criteria: aged over 18 years and proficient in Swedish. Exclusion criteria: individuals with relevant healthcare education or professional status. The decision to exclude these individuals was taken in order to minimize any individual’s authority affecting the group dynamics. Participants were recruited by MA in August–September 2016 through an area-based approach and purposive sampling, aiming for as heterogeneous groups as possible regarding gender, age, and education level [18]. Participants received a gift card of approximately EUR 25 after participating.

The FGDs were held in a meeting room at Uppsala University during October–November 2016. A female and a male senior lecturer, TG and SE, conducted the FGDs in Swedish and SE took notes. Participants were informed about the topic of the discussion. They had no prior relationship with the interviewers. The FGDs lasted between 90 and 120 min, including a break. After 30–40 min, participants watched a short video presenting basic facts on AR [19]. Data saturation was reached after three FGDs. The interviews were audio recorded and transcribed verbatim. No dropouts occurred.

### Data analysis

Data were analyzed using a directed approach to qualitative content analysis [20], in QSR International’s NVivo 11 Software. The HBM key constructs were used for the analysis (see Table 1). MA and TG analyzed the transcripts independently, compared outcomes and discussed inconsistencies. All authors discussed the results critically in frequent debriefing sessions and the study was also subjected to peer scrutiny and an audit trail. The Consolidated criteria for reporting qualitative studies (COREQ) was adhered to [21].

## Results

Twenty-three members of the general public participated in four FGDs (see Table 2). All participants were recruited from Uppsala city and areas nearby.

**Table 2:** Demographic information for the 23 participants

	G 1	G 2	G 3	G 4	
Woman	4	4	3	2	13
Man	3	2	3	2	10
Age					
Minimum	20				
Mean	38				
Maximum	81				
Education^a^					
EQF 4–5	12				
EQF 6–7	8				
EQF 8	3				
Reported history of antibiotic consumption					
Never taken	5				
Taken at least once	18				
Taken last year	4				
Taken before last year	14				

^a^Education was measured as the European Qualifications Framework (EQF) level. EQF 4–5 indicates high school, vocational school and university diplomas, EQF 6–7 indicates bachelor’s degree, vocational universities, and master’s degree, EQF 8 indicates doctoral degree

The results are presented according to the HBM. Quotes in Table 3 are used to provide evidence of authors’ interpretations, to offer readers greater depth of understanding, and to give research participants a voice. They are abbreviated as Q1, Q2 etc. in the text below.

**Table 3:** Exemplar quotes from the FGDs

Categories	Exemplar quotes	Group, participant
Perceived seriousness	Q1: “But I think it’s a bit like climate change also in the sense that it’s not so urgent ... you do not notice the changes now or so, the threats now, but ... when it breaks out… [then] one may regret it or realize that it is something important. So, it’s not like a tsunami in the sense that it’s immediate.”	G1, W4
	Q4: “…I know too little about these multi-resistant (bacteria), but you’re afraid of it, afraid to get them and suffer yourself, and afraid that… what would it be like if I couldn’t take any antibiotics?”	G1, W3
Perceived susceptibility	Q2: “By contrast, in other countries ... I know quite a lot of people abroad ... they take antibiotics several times a year. That’s where I feel the problem lies, perhaps not really in Sweden, but in what the others do.”	G3, W3
	Q3: “I’ve thought of this as a reason to just buy Swedish meat because it feels like it’s more controlled and it’s more certain that there are no drugs left.”	G1, M1
Perceived benefits	Q6: “To use them right when they really need to use them, that they don’t do it unnecessarily so they don’t get any type of anxiety or kind of negative feelings when it’s right to do it.”	G2, M1
	Q7: “Yes, it feels like a good compromise. I go abroad but I vaccinate first. Everyone is happy.”	G2, W3
Perceived barriers	Q5: “... then one pumps up antibiotics more and more when it may be possible to cure in another way, but because it works so well, one takes antibiotics and it has become natural to take them on many occasions ... and therefore it has become overused, actually. It has become something negative for something that has been very good.”	G1, W1
	Q8: “To put society’s best before oneself becomes harder and harder.”	G1, M3
	Q9: “…too easy to take, from the perspective of being too easy for both a doctor who is a bit fed up with his job and the patient who wants to recover quickly.”	G1, W2
	Q10: “[M]y basic problem with antibiotics, it’s still that no one in the world takes responsibility right now, it seems, and then it doesn’t matter how much we do in Sweden ... well, a little bit but it’s kind of minimal.”	G3, W3
Perceived self-efficacy	Q11: “But if you have such responsibility, it kind of includes some kind of sacrifice… For example, Thailand is a very popular destination now at Christmas. But Thailand is one of the premier sources of antibiotic resistance in the world; you should not really go there if one takes this somewhat seriously.”	G4, M1
	Q12: “It is difficult to be the one who refrains or stands by the one who refrains, but that ... yes, it is absolutely necessary.”	G1, M3
Cues to action	Q13: “...some global agreement, because then you get more encouraged. […] Feeling that it does matter the little I do…”	G1, W2
	Q14: “Then we of course should… like you state, discipline ourselves in society not to demand… to the same extent ask for antibiotics as soon we get nauseous or get a cold and so on. So I believe we are obliged to, as you say, enlightment, to inform, influence people. You can influence in many ways, not just through repetitive TV commercials, but maybe in many other informative ways.”	G3, M2

Quotes are labelled with G and 1–4 for the group number, W or M for the gender and 1–4 for the participant’s numerical code

### Perceived seriousness of and susceptibility to AR-related health issues

All particpants identified AR as a far-reaching health problem which could have terrible future consequences. Participants abundantly resorted to metaphors and analogies to describe the AR problem. An often recurring simile was climate change, compared with regard to the likelihood of being affected and its seriousness (Q1).

Although all participants acknowledged the threat, a kind of individual detachment was sometimes detected, as if only other people could be affected. Moreover, the doubt was sometimes voiced that individual judicious behavior might be futile. Participants harbored the idea that living in Sweden made it less likely that they would be affected by MDR bacteria (Q2). Accordingly, the perceived susceptibility was greater in relation to visiting countries with severe antibiotic resistance records; most mentioned was Thailand, which is a Swedish favorite holiday destination. Food, typically meat, was probably the most feared source of MDR bacteria, and often the dichotomy between Swedish and imported food was brought up (Q3).

Respondents frequently framed their fears in terms of possible suffering because of AR and that they may go untreated in the future because of extreme measures that could be taken to preserve antibiotic efficacy (Q4).

### Perceived benefits and barriers

The most important benefit of engaging in judicious use of antibiotics was the preservation of antibiotic effectiveness, both for the individual and the public. This concern also extended to future generations. This positive view of antibiotics comes with a risk that people get “spoiled” and demand antibiotics to great quantities or when it is not really necessary, the respondents pointed out (Q5). Many participants identified compliance with prescriptions and restricting personal use of antibiotics as important measures that would benefit individuals. This would lower the risk of there being no available treatment in the future and prompt the body’s immune system to respond, thus helping to withstand infections. Using fewer antibiotics, refraining from asking for antibiotics, and compliance with prescriptions, would also place “society first”. If one engages in judicious behavior, one can still use antibiotics when it is necessary, without feeling guilty about it (Q6). In the same way, one should take care to get vaccinated when traveling to countries with high AR records (Q7).

Considering the barriers, judicious behavior when e.g. consuming or travelling can conflict with individual interests as it can involve both individual efforts and costs (Q8). Other barriers were a perceived lack of international commitment to the fight against AR and overgenerous prescribing (Q9). Participants reported they had been able to easily obtain antibiotics while abroad and many viewed Sweden as being isolated in trying to act more judiciously (Q10).

### Self-efficacy in engaging in judicious behavior and potential cues to engagement

Participants expressed their willingness to engage in judicious behavior. They often gave altruistic reasons and believed that they had duties as individuals to so act (Q11). They reported good levels of perceived self-efficacy to engage in judicious behavior but still thought that it might become difficult (Q12). Such engagement was deemed appropriate only up to the point where life was threatened.

As to cues to action, participants stated that they would be more encouraged if their efforts would be part of a broader, international plan (Q13). It was also a general opinion that it would be beneficial to involve the public and that public awareness could be improved. Participants were quite aware of AR and of the fact that one should not misuse antibiotics, but were also markedly insecure about AR mechanisms, potential sources of MDR bacteria, and had many questions concerning antibiotics use. It was agreed that more communication from health authorities is needed, but there were contrasting opinions on the form it should take. A few participants stated that information about AR should be frightening (otherwise people would not take it seriously), but the majority felt that a more neutral form of communication would be more productive. Respondents trusted their physicians but had misgivings about communicating with healthcare personnel. They valued being informed as it is empowering; one can more easily make informed choices and also be more accepting when physicians do not prescribe antibiotics (Q14).

## Discussion

**Fig. 1:**
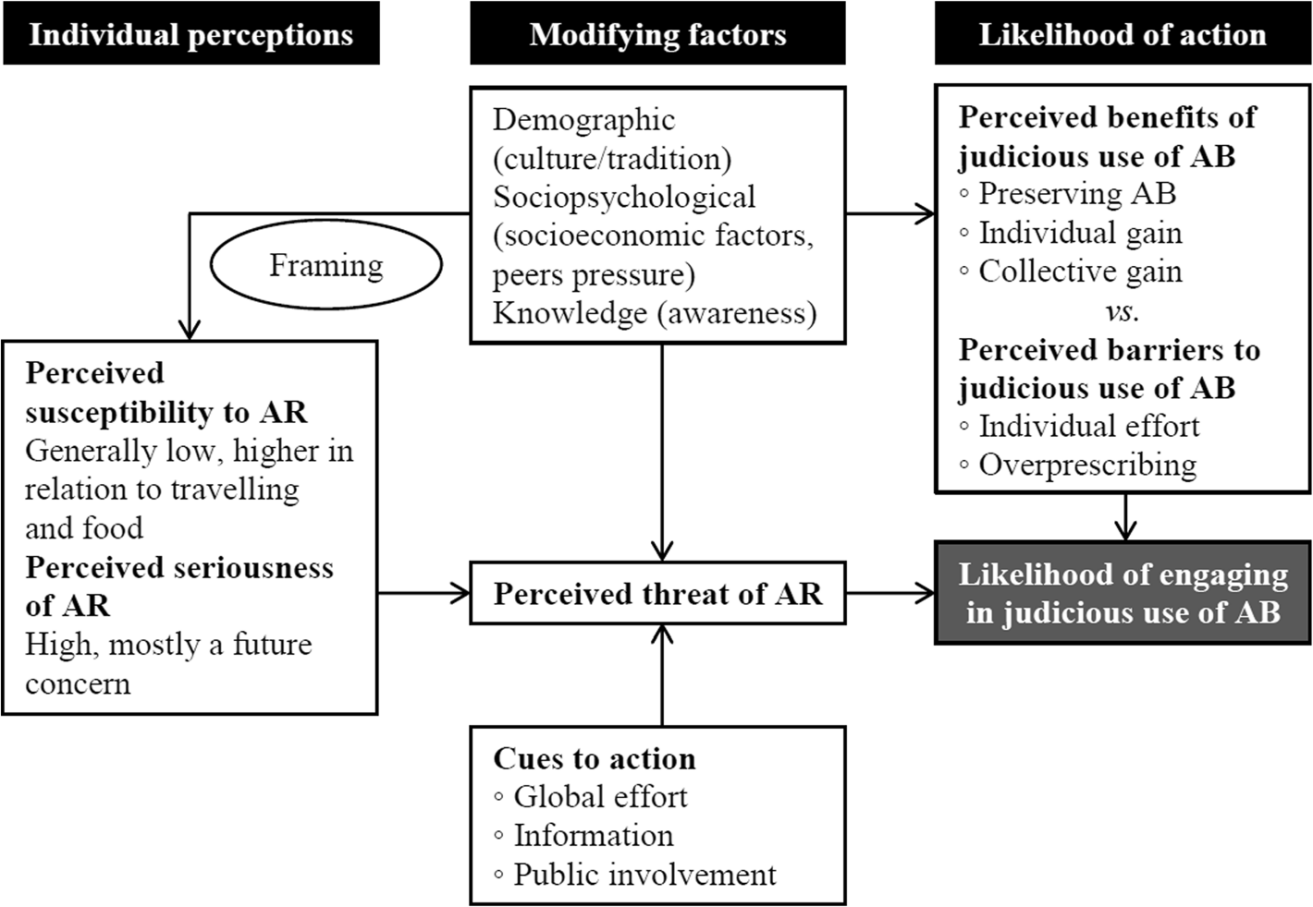
Results concerning AR and antibiotics (AB) use mapped through the HBM

The most striking results (see Fig. 1) were the prevalent description of AR as a slowly emerging problem that is somehow creeping up on us; the presence of a distinct tension between individual and collective interests; and the perceived need for empowerment through good health communication.

### A global problem sneaking up on us

The likelihood of being personally affected by AR was not perceived as high, yet participants were scared by the prospect of a future without effective antibiotics. This discrepancy between perceived seriousness of and perceived susceptibility to AR has previously been reported [11, 22]. Research in risk perception indicates that lay people consider the severity of materialized risks as more important than the likelihood of being affected because they perceive risks differently from experts: They have a broader notion of risk, incorporating considerations such as uncertainty, dread, and future generations [23]. Such regards might possibly be affected by the manner in which the situation is framed, as that influences how people understand risk [24]. The particular framing favored by the participants was climate change: a serious threat whose presence is not widely noticed but which is nonetheless progressing until it may be too late to remedy, a ‘slowly emerging disaster’.

It has been observed that apocalyptic narratives to describe the AR problem would be unsuitable for giving information about AR to the general public [25, 26]. Viens and Littmann [26] list some problematic issues and we found, in effect, support for these in the FGDs. Firstly, because of the proliferation of disaster language, its use could induce ‘disaster fatigue’ in the public, rendering the communication less effective. Secondly, talk of disasters can evoke ideas of the extraordinary use of severe restrictive measures on antibiotics. Thirdly, there is a risk that discursive overbidding about AR might induce fatalism or fears, which could stifle behavioral change.

Moreover, when the public needs to consider uncertain, future situations, disaster framing can induce responses such as a refusal to believe and misbehavior perpetuation [27]. Indeed, the participants expressed uneasiness about the vagueness of the threat and the uncertainty of when the disaster would become concrete. In contrast, the perception of the likelihood of being affected by AR increased when they discussed traveling and food, as the link between actions and potentially harmful consequences was easier to detect and more tangible. Also from this perspective, then, giving suitable information proves important.

### Individual and collective interests at odds

The participants were not purely self-interested. However, tension between expected individual gains and society’s needs emerged from the analysis of the benefits of and barriers to engaging in judicious behavior. It is known that antibiotics are thought of in extremely positive terms [28], so it is not surprising that the main drivers of participants’ overuse and misuse of antibiotics were the positive aspects of their use.

Another major, individualistic barrier to better behavior among participants was financial: To stay sick at home longer without resorting to antibiotics, or buying more expensive meat, entails an economic loss, which was discouraging for some participants. In Sweden, patients try to recover as soon as possible from illness and go back to work [29], and parents encourage their children to do the same [30]. Welfare policies, such as a more generous temporary parental benefit, could help to overcome these types of economic barriers [29].

Furthermore, we need to strengthen individual duties that could override such economic concerns. In the FGDs, values and norms supporting such obligations were found. There was a consensus in the FGDs that individual responsibility for public health provides a fundamental reason to modify non-judicious behavior. This was also expressed in moral terms. This finding of individual responsibility diverged from observations of other empirical studies, where the responsibility for tackling AR was largely or exclusively attributed to others, typically healthcare staff and authorities [22, 31]. However, it is consistent with the Swedish public health program where individual behavior and choice goes together with solidarity and the notion that people depend on one another [32].

Conceptually, it is useful to distinguish between egoistic and altruistic reasons for judicious behavior. As egoistic reasons, participants noted possible individual benefits such as a stronger immune system and reduction of the ‘no available treatment’ risk. Engaging in judicious behavior would also entail benefits such as being aware of contributing to the collective good and being protected against the shame of being responsible for non-judicious behavior. Research in fields such as HIV prevention and cancer clinical trials has highlighted the important role played by altruistic motivations [33, 34], but altruism is deficiently accounted for in the HBM as it rests on the assumption that health-related behavior should result from one’s own health concerns. However, previous studies confirm our finding, particularly those on willingness to vaccinate [35, 36], which have shown that individuals can engage in positive health behavior because of altruistic beliefs about a health threat to others.

### Health communication should empower

One particularly interesting and encouraging finding is that the partakers largely agreed on the need for multi-stakeholder involvement and responsibility. Not only do statutory bodies and clinicians carry duties to improve the situation; individuals have them too. The FGD participants stressed that the public should be involved and play a role. Therefore, the public should be further informed about AR and get involved in the work to counteract it. These findings are consistent with the Special Eurobarometer 2013 in which 85% of the Swedish population agreed that everyone has a role to play to reduce the risk for human health derived from antimicrobial resistance, compared to the EU average of 79% [6].

Individual responsibility involves patients understanding what to ask for when seeing the physician or other healthcare professionals, as pushy patients are often said to negatively influence prescribers’ behavior [4, 37]. While previous studies have shown that patients who obtain prescriptions for antibiotics tend to interpret these as representing quality of healthcare or concern for the patient [38], the FGD participants described overgenerous prescribing primarily as another barrier. Patients do want to act responsibly, but need support from ‘the system’ to become aware of what they should do. A reason for overgenerous prescribing (as some participants proposed) could be that family physicians give patients what they want, i.e. antibiotics, because they are worried about losing their patients to their colleagues [28, 38].

Despite the efforts carried out in Sweden since the 90s, FGD participants felt that more information is needed. Previous quantitative studies on the Swedish population confirm good levels of public awareness, but also found common confusion about antibiotics use and AR mechanisms and spread [8, 9]. A study on Swedish travelers found that low level of knowledge of antibiotic-resistant bacteria and the spread of resistance influenced travelers’ behavior and risk-taking, resulting in unaware exposure to risk situations [10]. The main focus of the FGDs was the need for accurate AR information from family physicians. Receiving accurate information would further individuals’ perception of self-efficacy and thereby empower patients to do the right thing, it was believed. This is in accordance with the literature, where there is substantial evidence for the association between effective communication, self-efficacy, and health behavior [39].

Interestingly, participants stressed how demotivating it was for them to think about countries without antibiotic control strategies and a lack of international coordination. What they read and hear seems to downplay what is actually achieved on an international level and emphasizes the problems abroad. Initiatives such as the EU One Health Action Plan against antimicrobial resistance, the global action plan against AR which was endorsed at the Sixty-eighth World Health Assembly in May 2015 [2], or that Sweden itself hosts the European Centre for Disease Prevention and Control (the main EU surveillance system on antimicrobial resistance) and also is the base of ReAct (an international network working on the containment of antibiotic resistance since 2005) [40, 41] largely go unnoticed. The key lesson here is that this lack of awareness of what is done to counteract AR seems to work as a powerful barrier to individual action, and any educational program should be careful to point to cooperative initiatives and give examples of successful programs. This would potentially work as an important cue to action.

### Limitations

This study has some limitations. The small sample was relatively homogeneous with mostly Swedish-speaking middle class from an urban area. Thus, the results might not be transferable to other populations and contexts, particularly those in a rural community or those with other cultural or ethnic diversity or social class. Therefore, we welcome more studies that attempt to replicate our study in a different setting or context.

## Conclusions

While lay people strongly and immediately perceive the severity of AR-related health issues, the way the AR problem is framed can influence perceived susceptibility negatively and hinder judicious behavior in relation to antibiotics use and AR. In communicating or engaging with the public, it should therefore be emphasized that AR is a significant public health issue that is already present and is getting worse. To think of it as some kind of future dilemma could lessen individual responsibility, which may reflect negatively on individuals and society. In addition, giving positive examples of ongoing international efforts to curb AR could be an important cue to engage in judicious behavior, as much as the lack of such examples could be a substantial barrier. People need to know that such international endeavor exists and feel that their contribution matter.

People already trust their physicians and rely heavily on information received from them. This means that if people could trust their physicians to diligently prescribe antibiotics, to explain why they do what they do, as well as inform them on how to act against AR, being aware of doing well for themselves and others could work as a powerful cue to action.

## Additional file

Additional file 1: Interview guide. Structure and contents (questions and probes) of the interview guide. (DOCX 20 kb)

## Abbreviations

AB: Antibiotics
AR: Antibiotic resistance
COREQ: Consolidated criteria for reporting qualitative studies
FGD: Focus group discussion
HBM: Health Belief Model
HIV: Human immunodeficiency virus
MDR: Multidrug resistant
WHO: World Health Organization
