# Supplementary material for: Public awareness and individual responsibility needed for judicious use of antibiotics: a qualitative study of public beliefs and perceptions
Source: BMC Public Health. 2018 Oct 3;18:1153. doi: 10.1186/s12889-018-6047-8 (PMC6171135; doi:10.1186/s12889-018-6047-8)
Supplement: Supplementary file 1 — Interview guide. Structure and contents (questions and probes) of the interview guide. (DOCX 20 kb) [file 12889_2018_6047_MOESM1_ESM.docx]

# Interview guide

| **Opening question** | 1) What is your name and why did you decide to partake in this discussion? |
| --- | --- |
| **Introductory question** | 2) What is the first thing that comes up in your mind thinking about antibiotics? |
| **Transition question** | 3) What experience do you have with antibiotics? |
| **Key questions** | 4) Please write down a list of what you think of as advantages and disadvantages with use of antibiotics.  (Participants are invited to share their lists and discussion is promoted) |
|  | 5) Do you think that you should have the right to buy antibiotics by yourself without prescription?  Probe: What do the others think? |
|  | *- Break and short movie -* |
|  | 6) How would you react if you had a fever and a cough and the doctor says no about prescribing antibiotics to you?  Probing questions: Group reflection about whether this is perceived as dangerous, about buying antibiotics online and using leftovers. |
|  | 7) As you saw in the movie, there are risks related to antibiotic resistance. What do you think about it and why? |
|  | 8) Thanks to antibiotics, healthcare has had great success in treating infections. We know that increased resistance is of particular concern for groups at risk, such as immunocompromised patients, those who undergo major surgery, patients in cancer treatment, etc. For their sake it could be important that we all use antibiotics responsibly. What do you think about this?  Probing questions: Group reflection about whether responsible use is difficult/burdensome, about who is or should be held responsible and about future generations. |
|  | 9) Some researchers said: “The solution may ultimately require us to put society before the individual. That is, halting the rise of resistance may only be achievable if some patients go untreated”. Is this reasonable? |
|  | 10) What are you prepared to do to counter antibiotic resistance?  Probing questions: Group reflection about whether they could do more vaccines, travel less, and other actions. |
| **Ending question** | 11) If you could give a suggestion to policy makers about antibiotics use, what would be your advice? |
